# Nontraumatic First Rib Fracture in a Young Weightlifter Resulting in Winged Scapula: A Case Report

**DOI:** 10.5811/cpcem.49035

**Published:** 2026-04-29

**Authors:** Jessica Remy, Nicole Prendergast

**Affiliations:** Atrium Health Wake Forest Baptist, Department of Emergency Medicine, Winston-Salem, North Carolina

**Keywords:** shoulder pain, scapular winging, dorsal scapular nerve, first rib fracture, case report

## Abstract

**Introduction:**

Shoulder pain is a common emergency department (ED) presentation. Scapular winging is a rare condition often associated with long thoracic nerve injury.^9^ This case report describes an even rarer case of dorsal scapular nerve injury caused by a nontraumatic first rib fracture in a young weightlifter, an injury mechanism not previously reported in the literature.

**Case Report:**

A 17-year-old male presented to the ED with left shoulder pain following weightlifting. Physical examination demonstrated scapular winging, and a clinical diagnosis of dorsal scapular neuropraxia was made. Imaging revealed a nontraumatic first rib fracture. The patient was treated conservatively with nonsteroidal anti-inflammatory drugs and rest, resulting in complete resolution of symptoms within two weeks at clinic follow-up.

**Conclusion:**

To our knowledge, this is the first case in the emergency medicine literature of a nontraumatic rib fracture with associated dorsal scapular nerve injury and scapular winging. This case highlights the importance of thorough visual inspection and movement assessment by emergency physicians in patients presenting with shoulder pain, as key findings such as scapular asymmetry and neurologic injuries may otherwise be missed. Recognizing rare injuries like nontraumatic first rib fractures with associated neurologic deficits is critical for timely diagnosis and management, which can lead to excellent outcomes.

## INTRODUCTION

Shoulder pain is a common presenting complaint in the emergency department (ED).^1^ While scapular winging is a rare diagnosis, it is an important clinical finding that can signify underlying neurologic or structural injury. Scapular winging most frequently results from injury to the long thoracic nerve, affecting the serratus anterior muscle. However, less common etiologies include injury to the spinal accessory or dorsal scapular nerves, which are rarely reported in the literature.^2^–^4^ The first rib, located in the cephalic thorax, is infrequently injured. Traumatic fractures typically result from significant blunt force, whereas nontraumatic first rib fractures are exceedingly rare and most associated with repetitive stress or muscle force imbalance. Adolescent athletes are particularly vulnerable due to the discrepancy between rapid muscle development and delayed bony maturation.^5^,^6^

We report a unique case of a 17-year-old male presenting to the ED with left shoulder pain and scapular winging secondary to dorsal scapular nerve injury associated with a nontraumatic first rib fracture. To our knowledge, this is the first reported instance of this combination of injuries. This case emphasizes the importance of visual inspection and movement assessment in patients presenting with shoulder complaints and highlights the potential for conservative management to achieve complete recovery in such cases.

## CASE REPORT

**Image 1:**
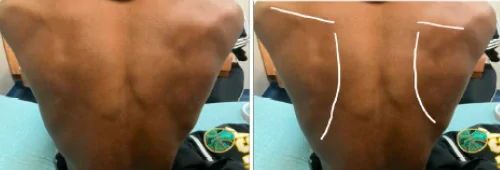
A) Visual inspection with upper extremities protracted; B) white line delineating approximate medial scapular border, demonstrating winging of the scapula in a teenage male whose pain began after performing deadlifts.

A 17-year-old male with no significant past medical history presented to the ED with five days of left shoulder pain. The pain began after he performed deadlifts in weightlifting class at school the day prior to symptom onset. He described pain in his posterior shoulder with no known trauma or injury. The patient’s mother observed an abnormal appearance of his left upper back, prompting the visit. On examination, visual inspection demonstrated protraction of the inferior aspect of the left scapula with resulting asymmetry consistent with scapular winging (Image 1). The patient was noted to have edema and tenderness on palpation to the posterior, inferior portion of the shoulder joint. Neurovascular examination showed intact sensation and pulses bilaterally, with 5/5 strength in all planes of motion. Special testing, including the empty-can test, was negative. There were no signs of shoulder droop or scapular elevation.

**Image 2:**
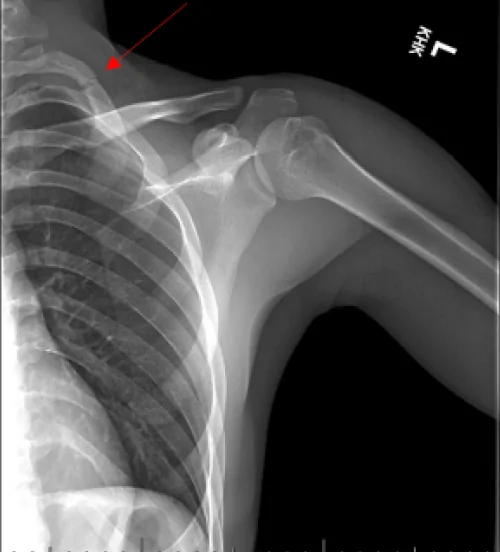
Scapula radiograph demonstrating incidental finding of first rib fracture marked by arrow, in the case of a teenage male injured while weightlifting.

Initial radiographs of the left scapula were obtained, which incidentally revealed a first rib fracture (Image 2). This was confirmed on subsequent chest and cervical spine radiographs. The shoulder was located without additional bony or soft tissue abnormalities.

A clinical diagnosis of scapular winging resulting from neuropraxia was made in the ED. This was attributed to stretching of the nerve caused by opposing muscle forces acting on the first rib during weightlifting, resulting in fracture of the rib. The patient was treated conservatively with a 14-day course of diclofenac 75 mg twice daily. At follow-up in the sports medicine clinic eight days after initial ED presentation, the patient demonstrated complete resolution of symptoms, including the scapular winging. He was advised to avoid lifting greater than 10 pounds and refrain from participating in contact sports for four weeks. Further follow-up was deemed unnecessary unless symptoms recurred.

## DISCUSSION

Scapular winging resulting from nerve injury is an uncommon finding in the ED. Most cases are attributed to long thoracic nerve damage affecting the serratus anterior muscle. The dorsal scapular nerve innervates the rhomboid muscles, which insert on the inferomedial scapula and are responsible for retracting and elevating its medial border. Injury to the dorsal scapular nerve is a rare cause of scapular winging and typically arises from nerve entrapment in the middle scalene muscle or excessive traction during repetitive activities.^4^,^7^,^8^ This case is unique in that the dorsal scapular neuropathy, a rare entity itself, was associated with a similarly uncommon nontraumatic first rib fracture.

*CPC-EM Capsule*

What do we already know about this clinical entity?

*Adolescent athletes are at high risk for overuse, stress and non-contact injuries due to bone growth and its impact on surrounding muscles, ligaments and tendons.*

What makes this presentation of disease reportable?

*This rare case of a nontraumatic rib fracture caused dorsal scapular nerve injury and scapular winging, with potential for long-term deficits if it had been missed.*

What is the major learning point?

*Exposure and careful visual inspection are essential in the evaluation of musculoskeletal complaints as asymmetry can reveal subtle neurologic injury.*

How might this improve emergency medicine practice?

*Understanding adolescent musculoskeletal injuries underscores the value of visual inspection in the physical exam.*

The first rib, due to its anatomic positioning behind the clavicle and shoulder muscles in the thorax, is seldom injured without significant trauma. Nontraumatic fractures are rare and primarily occur in adolescent athletes due to the disproportionate strength of developing musculature compared to immature bone.^5^ There are multiple muscular attachments to the first rib: the scalene; intercostal; and serratus anterior muscles. The scalene muscles exert a superior force on the rib while the intercostal and serratus anterior muscles attach on the inferior surface, pulling the rib downward with their contraction. The first rib’s subclavian groove, the narrowest portion, has been identified in literature as the most prone to fracture. The mechanism of injury in this case likely involved opposing forces of the scalene and serratus anterior muscles, described above, exerted on the first rib during a deadlift maneuver. This hypothesis is supported by previous case reports that have described similar mechanisms of nontraumatic first rib fractures in adolescent athletes, such as weightlifters and cheerleaders, due to sudden neck muscle contraction.^5^,^6^

However, neurologic injuries associated with first rib fractures are exceptionally uncommon, and dorsal scapular nerve involvement is particularly rare.^4^ Although, cases of first rib fracture and dorsal scapular nerve injuries have been previously described independently, none to our knowledge have reported a fracture with the concurrent presence of dorsal scapular neuropathy leading to scapular winging. A previous case report in a boxer described dorsal scapular neuropathy secondary to muscle imbalance between the serratus anterior and rhomboid muscles created by microtrauma resulting in stretching and nerve impairment.^7^ While injury to the scalene muscles can result in dorsal scapular nerve entrapment, we believe the fracture mechanism in this case reflects the reverse process—forceful contraction of the scalenes generating the first rib fracture.

We propose two prospective mechanisms for injury of the dorsal scapular nerve: traction-related stretch injury; and entrapment due to muscle hypertrophy or inflammation. Our patient exhibited significant scalene contraction during the lift, generating enough force to fracture the rib, potentially producing traction on the dorsal scapular nerve as it traverses the middle scalene. Additionally, local swelling or hypertrophy of the middle scalene after fracture may have led to dorsal scapular nerve entrapment (Table).^9^ Our patient’s scapular winging resolved with conservative management, including nonsteroidal anti-inflammatory drug therapy and rest, like the previous case described, which is consistent with a diagnosis of neuropraxia.^7^ In this case, we were able to make a clinical diagnosis of scapular winging due to dorsal scapular nerve pathology.

Based on our patient’s reassuring exam without evidence of neurovascular compromise, no advanced imaging was indicated in the ED. Rapid clinical recovery negated the need to pursue magnetic resonance imaging (MRI) in this patient. Persistent weakness or scapular dyskinesis at follow-up would warrant MRI and/or electromyography to evaluate for nerve compression or anatomic variants. In select cases, point-of-care ultrasound may assist with diagnosis—detecting rib fractures, assessing scalene hypertrophy, or visualizing dorsal scapular nerve entrapment. This innovative use of dynamic ultrasound has been described in adolescent athletes.^10^ Our patient had no history of direct trauma; therefore, computed tomography was not felt to be appropriate in this pediatric patient to minimize exposure to radiation.

This presentation underscores the need for careful inspection and movement assessment in the evaluation of ED patients with shoulder pain to identify rare, subtle findings like scapular asymmetry, which is likely under-reported and may indicate underlying neurologic or structural injuries.^11^ Failure to diagnose scapular winging or its etiology can predispose patients to further injury or muscle atrophy. The stability of the scapulothoracic joint depends on the coordinated function of the supporting musculature, and undiagnosed deficits can lead to secondary injuries of the glenohumeral joint.^12^ This case highlights the critical role of emergency physicians in identifying subtle physical exam findings and considering rare diagnoses in the evaluation of shoulder pain.

## CONCLUSION

Nontraumatic first rib fractures with associated neurologic injury resulting in scapular winging is a rare diagnosis. Adolescent athletes may be more predisposed to nontraumatic first rib fracture, which should be considered in any athlete presenting with shoulder pain. This case underscores the importance of the physical exam, including visual inspection at rest and range of motion assessment of the shoulder girdle and scapulothoracic joint as it can be essential for accurate diagnosis of scapular winging, a subtle indicator of underlying neurologic or structural injury. Emergency physicians must consider this fracture diagnosis in the appropriate adolescent athlete population. Scapular winging should also be considered in most others with nontraumatic shoulder pain to ensure timely identification and treatment. Early recognition and conservative management, as demonstrated in this case, can lead to excellent prognostic outcomes, preventing long-term complications such as muscle atrophy or secondary injuries to the shoulder joint.

## Figures and Tables

**Table:** Scapular winging nerve, muscle groups involved, scapular appearance, and differential in the case of a teenage athlete injured while weightlifting.

	Nerve involved	Muscle group involved	Scapular movement observed	Differential
Lateral scapular winging	Long thoracic nerve	Serratus anterior	Superior portion of the scapula moves laterally while tip of the scapula moves medially.	Primary neurogenic: Spinal accessory nerve palsy (most often iatrogenic after neck dissections/lymph node biopsy; also traction, blunt/penetrating trauma) Dorsal scapular nerve palsy with rhomboid weakness (entrapment at middle scalene, Cervical nerve 5 radiculopathy/traction injury Systemic/neuromuscular: Facioscapulohumeral dystrophy (relative weakness of trapezius/rhomboids/levator versus deltoid/rotator cuff).
Medial scapular winging	Spinal accessory nerve (cranial nerve XI), dorsal scapular nerve	Rhomboids, trapezius	Entire scapula moves laterally	Primary Neurogenic Long thoracic nerve palsy (traction/overuse, compressive, iatrogenic; eg, chest tube, thoracic/axillary surgery) Serratus anterior myopathy Parsonage-Turner/brachial neuritis Cervical nerve VII radiculopathy Brachial plexopathy. Systemic/neuromuscular: Facioscapulohumeral dystrophy; limb-girdle muscular dystrophies Inflammatory/viral neuropathies (eg, Guillain-Barré syndrome, Lyme disease, systemic lupus erythematosus associations). Structural and trauma: Serratus anterior avulsion Inferior pole scapular fracture
